# Bioactive Amines Screening in Four Genotypes of Thermally Processed Cauliflower

**DOI:** 10.3390/antiox8080311

**Published:** 2019-08-15

**Authors:** Marla Silvia Diamante, Cristine Vanz Borges, Mônica Bartira da Silva, Igor Otavio Minatel, Camila Renata Corrêa, Hector Alonzo Gomez Gomez, Giuseppina Pace Pereira Lima

**Affiliations:** 1 Department of Chemistry and Biochemistry, Institute of Biosciences, São Paulo State University (UNESP), Botucatu, São Paulo 18600-970, Brazil; 2 Departamento de Agronomia, Universidade do Estado de Mato Grosso (UNEMAT), Cáceres 78200-000, Mato Grosso, Brazil; 3 Department of Pathology, School of Medicine, São Paulo State University (UNESP), Botucatu, São Paulo 18600-970, Brazil; 4 Department of Food Technology, Universidad Nacional de Agricultura (UNAG), Catacamas 16021, Honduras

**Keywords:** *Brassica oleracea* var. *botrytis*, antioxidants, biogenic amines, cooking

## Abstract

Biogenic amines are important indicators of food quality with recognized antioxidant capacity. Diets that are rich in these compounds promote several benefits for human health, although the consumption in excess may result in food poisoning. This study aims to screen the levels of biogenic amines in four colored cauliflowers, before and after cooking (boiling, steaming, and microwaving). In addition, the levels of tryptophan and 5-hydroxytryptophan, two serotonin precursors, were analyzed. Our results reveal that thermal processing shows a tendency to increase tryptophan levels and reduce 5-hydroxytryptophan in colored cauliflowers. A reduction of the tryptophan and increase in serotonin contents in ‘Cheddar’, steamed or microwaved, was observed. A higher level of histamine was observed in the genotype ‘Forata’ after cooking, whereas melatonin levels were higher after steaming and microwaving. The lowest levels of biogenic amines and amino acids were observed in ‘Graffiti’. All the colored cauliflowers that were analyzed presented a chemical quality index (CQI) below the pre-established limits, indicating that are safe for consumption, even after cooking. We conclude that the levels of biogenic amines and amino acids in colored cauliflower are safe for human consumption and do not present health risks. Therefore, the consumption of these genotypes, raw or cooked, is a good source of bioactive compounds.

## 1. Introduction

The food consumption is change, and the concern for foods with more nutritional quality is evident. Health behaviors, like eating different vegetables, are associated with diseases prevention and contributing to physical and emotional wellbeing [1]. The protective mechanism by which vegetables protects or improve the human body response to oxidative stress and inflammation include the action of their bioactive compounds [2]. Traditional cauliflower or colored genotypes are a good source of antioxidants that may be interesting to protect against oxidative stress and inflammation processes present in chronic diseases.

Biogenic amines are compounds ubiquitous in all food and may show direct action on the organisms, due to its antioxidant function [3]. In foods, some of the most common biogenic amines are putrescine, cadaverine, spermidine, spermine, histamine, tyramine, tryptamine, and agmatine [4]. However, serotonin and dopamine have been described in fruits and vegetables [5,6], and some amines are a very powerful antioxidant.

The serotonin is widely distributed in the vegetal kingdom [7], and some studies demonstrate that it is a powerful antioxidant and radical scavenger. It also can be used as a biochemical marker in some stages of plant development [8]. In mammals, the serotonin is a neurotransmitter that acts in the central nervous system, affecting appetite, sleep, anxiety and humor [1]. In humans, serotonin is produced from the tryptophan by the action of the tryptophan 5-hydroxylase (EC 1.14.16.4), which converts it in 5-hydroxytryptophan. Then, this compound is decarboxylated by the tryptophan decarboxylase (EC 4.1.1.28), in order to form serotonin [9]. In plants, the synthesis of serotonin occurs mainly by the tryptamine biosynthesis pathway. The tryptophan is initially converted in tryptamine by the action of the tryptophan decarboxylase (EC 4.1.1.28), and it is converted in serotonin by the action of tryptamine 5-hydroxylase [10,11].

In plants, the tryptophan is related to the growth, due to the formation of auxin, and also due to the formation of such defense substances, as glucosinolates and alkaloids [12,13]. The tryptophan is an amino acid essential for humans and is obtained by the ingestion of eggs, milk, meat, soy, potatoes, broccoli, cauliflowers, eggplant, bananas, among others [14]. Most of tryptophan is converted in signaling molecules of the neuroimmunological processes, and less than 1% is used in the biosynthesis of proteins [1].

As well as serotonin, dopamine is a neurotransmitter related to the sensation of pleasure and satisfaction. Both of them play an important role in neuropsychiatric disturbs, such as depression, schizophrenia, and Parkinson disease, regulating specific paths [1,15]. In plants, dopamine plays an important role in the intracellular regulation of permeability and photophosphorylation, due to its free radical scavenging ability. Dopamine is considered a strong hydrosoluble antioxidant with higher antioxidant capacity than the glutathione, catechin, quercetin and luteolin, with a capacity similar to the antioxidants gallocatechin and ascorbic acid [6,16], thus protecting the growth and the productivity of plants under different stress conditions [17].

Melatonin is produced from the serotonin. In mammals, the melatonin acts mainly in the physiological processes related to the regulation of circadian rhythm, humor and sleep [3]. Plants may present a higher quantity of this substance when compared to animals. In plants, the melatonin acts as an antioxidant, affects the vegetal growth, has the function of stabilizing the cellular membrane and regulates the gene expression [9]. In addition, it has been associated with the reproductive development (flowering), including the circadian rhythms (photoperiod) [18] and as a signaling molecule in the mechanism of defense against pathogen and others biotic and abiotic stresses [19].

Other amines, such as putrescine, spermine and spermidine are related to cellular processes, directly involved in the growth, division and cellular differentiation processes [20]. These amines, together with the histamine and cadaverine, are used as a quality parameter of some foods through the CQI (Chemical Quality Index) [21]. Even though do not exert any direct toxic effect, some amines as putrescine, spermidine and spermine can potentiate the harmful effects of histamine by competing for the detoxifying enzymes and acting as precursors of the nitrosamines [20].

Some brassicas, as the cauliflowers, present different levels of biogenic amines [22,23], are rich in bioactive compounds and present low calories, which contributes to the preference of the consumers for this vegetable. Traditionally, the white genotype is the most consume—however, other colored genotypes, such as the purple, green and orange, can be an option for the consumption. These genotypes are well-established and produced in several countries in the world [24]. Generally, the consumption of this vegetable is performed after the thermal processing, which can affect the content of some bioactives, including the biogenic amines [25]. The cooking facilitates the extraction of some metabolites of vegetal matrix [26,27]—however, it can promote losses of nutrients, due to the leaching and cellular destruction [20,25]. The biogenic amines are not considered thermostable [5], and studies demonstrate that the method of cooking and the employed time can affect the levels of these substances promoting an increase or decrease [20,25], depending on the food matrix. 

This study demonstrates the effects of the cooking process (boiling, steaming and microwaving) and time in the levels of amino acids (tryptophan and 5-hydroxytryptophan) and biogenic amines of colored cauliflowers. 

## 2. Materials and Methods

### 2.1. Plant Material

Four colored cauliflowers genotypes—‘Forata’ (white inflorescence), ‘Verde di Macerata’ (green inflorescence), ‘Cheddar F1’ (yellow inflorescence), and ‘Graffiti’ (purple coloration) were obtained from a local producer and carefully selected to avoid plants with visible damage in the inflorescences. 

The florets of each genotype with approximately five centimeters were washed in tap water and immersed in sodium hypochlorite solution (100 mg/L). The raw material was divided into 300 g portions. One part was retained raw and other portions were submitted to the following cooking treatments: Boiling (baking at ≅ 95 °C in stainless steel pan containing 1000 mL of water), steaming (baking at ≅ 95 °C using stainless steel pan containing 800 mL of water) or microwaving (baking at domestic microwave—medium power, using glass refractory recipient, containing 100 mL of water and covered with plastic film). Each thermal processing was done by 5 and 10 min, and after that separated into subsamples (*n* = 3), lyophilized (Lyophilizer Terroni Scientific, São Carlos, Brazil, model LD1500), and grounded to a fine powder to storage at −80 °C until analyzes. 

### 2.2. Biogenic Amine Analysis by HPLC

The biogenic amines in florets of colored cauliflowers were extracted as previously reported [28]. Lyophilized samples were homogenized in 3 mL of 5% perchloric acid (HClO_4_), held in an ultrasonic bath for 30 min and centrifuged at 6000 rpm for 10 min (5 °C). In 200 μL of the supernatant were added 400 μL of dansyl chloride (2.5 mg/mL acetone) and 200 μL of the saturated sodium carbonate solution. After stirring and one hour remained in the dark at 60 °C, 200 μL of proline (99%) (0.1 mg/mL in ultrapure water) were added. The mixture was maintained at room temperature for 60 min, and toluene (1000 μL) was used to extract the dansylated PAs. The samples were vortexed for 1 min, and the supernatants were withdrawn and subjected to drying with N_2_ and resuspended in 1 mL of acetonitrile HPLC (High Performance Liquid Chromatography) grade (assay 99.9%). Then, the mixture was kept in an ultrasonic bath for 1 min and centrifuged for 5 min at 4000 g (4 °C). The supernatant was filtered (0.22 μm) before injection into a UHPLC (Ultra High Performance Liquid Chromatography). The chromatographic separation was performed using a Dionex Ultimate 3000 Thermo Scientific system (Thermo Fisher Scientific Inf.; Santa Clara, CA, USA) coupled to a quaternary pump, an automatic sampler (model 3000RS) and a diode arrangement detector (DAD—3000RS). Twenty microliters of sample were injected and the chromatographic data were collected and processed using Chromeleon 7 software (Thermo Fisher Scientific, Bremen, Germany), at a flux of 0.7 mL/min, using an Ace 5 C18 (Advanced Chromatography Technologies, Aberdeen, UK) column (4.6 mm × 250 mm, 5 μm particle size) at 25 °C. The detection was adjusted to 225 nm, and the peak integration and calibration were performed between 225 and 300 nm. The chromatograph gradient was established to a mixture of solvents (A) 100% acetonitrile and (B) 50% acetonitrile, as follows: 0–2 min, 40% A + 60% B; 2–4 min, 60% A + 40% B; 4–8 min, 65% A + 35% B; 8–12 min, 85% A + 15% B; 12–15 min, 95% A + 5% B; 15–21 min, 85% A + 15% B; 21–22 min, 75% A + 25% B; 22–25 min, 40% A + 60% B. Identification and quantification of amino acids (tryptophan and 5-hydroxytryptophan) and biogenic amines (tryptamine, serotonin, melatonin, dopamine, histamine, cadaverine, putrescine, spermidine and spermine) were based on their retention times, compared to the standards of each compound. The results were expressed as μg/g of dry weight.

### 2.3. Chemical Quality Index—CQI

The CQI was estimated from the biogenic amines related to the food deterioration process [21], using the total concentration (ug/g d.w.) of histamine (cHIM), putrescine (cPUT), cadaverine (cCAD), spermidine (cSPD), and spermine (cSPM), and calculated with the following Equation (1):

(1) $$\mathrm{CQI}=\frac{\mathrm{cHIM}+\mathrm{cPUT}+\mathrm{cCAD}}{1+\mathrm{cSPD}+\mathrm{cSPM}}.$$

### 2.4. Statistical Analysis

All samples were analyzed in triplicate and submitted to a one-way analysis of variance (ANOVA), followed by the Scott-Knott average comparison test (*p* < 0.05). The experimental results were expressed as the mean ± standard deviation (mean ± SD). The principal component analysis was carried out using the XLSTAT software—version 2017 (Addinsoft, Paris, France).

## 3. Results and Discussion

**Figure 1:**
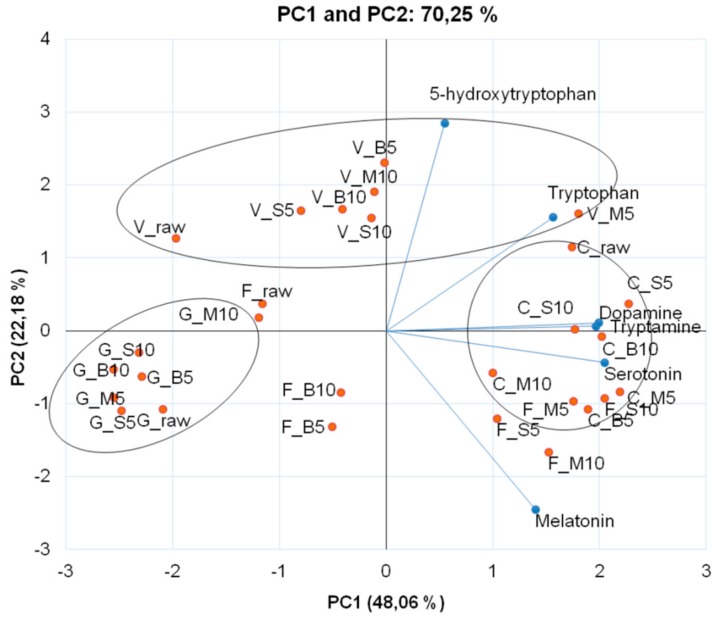
Bi-dimensional projection and scores of the amino acids tryptophan and 5-hydroxytryptophan, and the biogenic amines (serotonin, tryptamine, melatonin and dopamine) in thermally processed colored cauliflowers. The treatments are represented by the points, where initial letters represent the genotypes (V = ‘Verde di Macerata’, G = ‘Graffiti’, F = ‘Forata’ and C = ‘Cheddar’) and the letters after underline represent the cooking methods (raw, B = boiling, S = steam and M = microwave) and the numbers represent the cooking times (5 min and 10 min).

In order to establish a descriptive model with the levels of serotonin and melatonin, as well as their precursors tryptophan and 5-hydroxytryptophan, in colored cauliflowers after thermal processing, we opted to use the principal component analysis (PCA) for better visualization of the results. PC1 (principal component 1) and PC2 (principal component 2) explained 70.25% of the data set variance. Both the precursors (tryptophan and 5-hydroxytryptophan), as well as serotonin and melatonin, occurred in PC1+ (principal component 1, positive quadrant), which represents 48.06% of the total variation data, separating the genotypes and the thermal processes applied (Figure 1).

**Figure 2:**
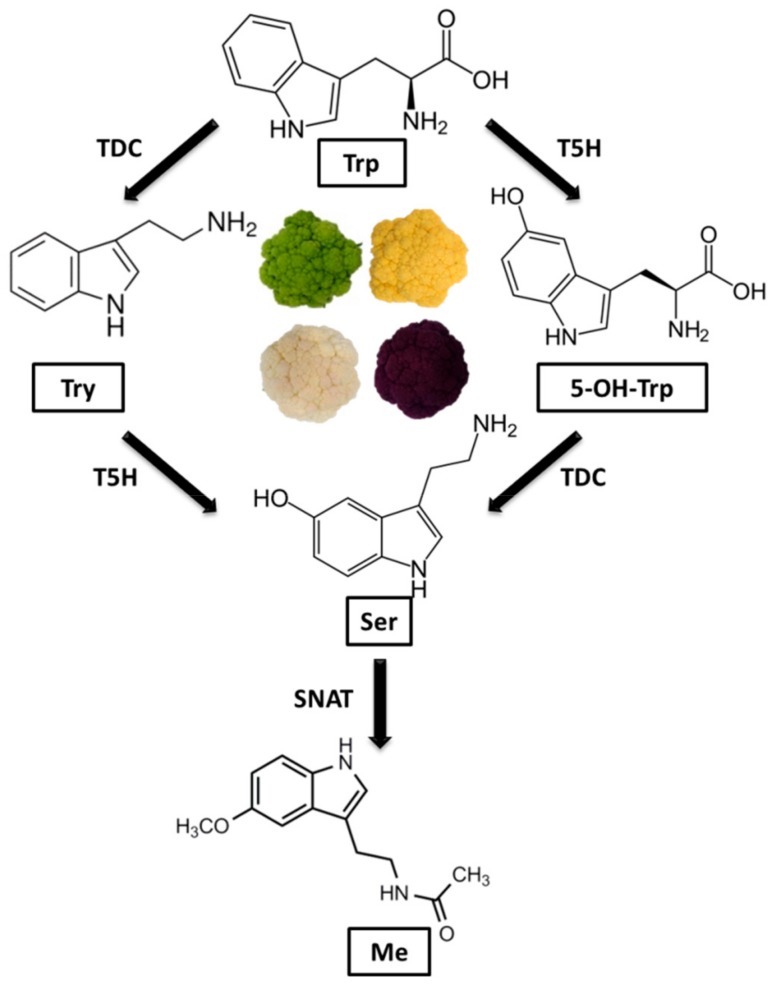
Flowchart evidencing the pathway of serotonin from tryptophan in raw colored cauliflowers. Trp—tryptophan; Try—tryptamine; 5-OH-Trp—5-hydroxytryptophan; Ser—serotonin; Me—melatonin; T5H—tryptophan 5-hydroxylase; TDC—tryptophan decarboxylase; SNAT—serotonin N-acetyltransferase.

‘Cheddar’ raw inflorescences standout in relation to the levels of tryptophan (337.35 μg/g d.w.), 5-hydroxytryptophan (384.97 μg/g d.w.), tryptamine (9.00 μg/g d.w.), melatonin (5.33 μg/g d.w.) and dopamine (0.42 μg/g d.w.) (Table 1). On the other hand, raw ‘Forata’ inflorescence showed the highest level of serotonin (0.87 μg/g d.w.). The high level of tryptophan and low tryptamine may be attributed to the reduced activity of the enzyme tryptophan decarboxylase (TDC) in the four genotypes of cauliflower (Figure 2), which is able to convert tryptophan into tryptamine. The preferred pathway of serotonin synthesis in plants is through the hydroxylation of tryptamine, by the enzyme tryptophan 5-hydroxylase (T5H), to produce serotonin [11]. In animals, the serotonin synthesis occurs in a reversed step, with 5-hydroxytryptophan being produced by tryptophan hydroxylase and then being decarboxylated, by aromatic acid decarboxylase, to form serotonin [9]. In our samples, a strong correlation was found between tryptamine and tryptophan (*R*^2^ = 0.92) only in the ‘Forata’ genotype. However, in the other genotypes this correlation was weak (‘Verde di Macerata’ *R*^2^ = 0.50; ‘Cheddar’ *R*^2^ = 0.13; ‘Graffiti’ *R*^2^ = 0.20), evidencing the need for more detailed studies of these pathways in colored cauliflower. It is noteworthy that the considerable amounts of 5-hydrohytryptophan found in colored cauliflowers may be interesting to consumers, once this amino acid is a precursor of serotonin [9].

All the thermal processes promoted increases of tryptophan levels in up to four times in relation to raw in ‘Verde di Macerata’ (Table 1). In the raw ‘Cheddar’, which contains high levels of tryptophan (337.35 μg/g d.w.), only the boiling induced alterations in the contents of this amino acid in both cooking times, i.e., at 5 (362.25 μg/g d.w.) and 10 (399.40 μg/g d.w.) min. On the other hand, raw ‘Graffiti’ contains the lowest levels of tryptophan (21.29 μg/g d.w.), and the thermal processing was able to increase the tryptophan content. Tryptophan is a precursor of serotonin and melatonin [13], and it is essential for the formation of auxins [29] and defense compounds [19], such as indole glucosinolates and indole alkaloids in plants [12,13].

As aforementioned, the tryptophan ingestion is essential to maintain normal body composition and function. According to World Health Organization (WHO), the tryptophan requirement range from 9.5 mg/kg per day in children aged 0.5 (years) to 4.0 mg/kg per day in adults (> 18) [30]. Vegetables as potatoes (64.47 mg/kg f.w.), onion (36.47 mg/kg f.w.), broccoli (36.12 mg/kg f.w.), spinach (32 mg/kg f.w.), melon (30.77 mg/kg f.w.), and banana (26.15 mg/kg f.w.) are a good source of tryptophan [23]. Nevertheless, the ingestion of ‘Cheddar’ or ‘Verde di Macerata’ inflorescences after cooking for 10 min, either by boiling, steam or microwave, can provide around 40 mg of tryptophan, which together with other foods rich in that amino acid could contribute to obtaining a healthy diet. 

5-hydroxytryptophan and tryptamine levels were altered in the colored cauliflower after cooking. The highest levels of 5-hydroxytryptophan were noticed in raw inflorescences of ‘Cheddar’ (384.97 μg/g d.w.) and ‘Verde di Macerata’ (254.39 μg/g d.w.) and the cooking induced a decrease in the content. ‘Graffiti’ inflorescence (raw = 42.90 μg/g d.w.) after cooking for 10 min showed an increase in the levels, either by steam (50.49 μg/g d.w.) or microwave (77.13 μg/g d.w.) (Table 1). The levels of tryptamine decrease in ‘Cheddar’ after thermal processing. The raw ‘Cheddar’ cauliflower presented higher tryptamine content (9.00 μg/g d.w.) in relation to the other raw genotypes and in addition, all the cooking methods for 5 min induced an increase in the levels of this compound.

The intake of tryptophan and 5-hydroxytryptophan is essential for the formation of serotonin in the brain because this neurotransmitter does not cross the blood-brain barrier and the synthesis and turnover of serotonin depends on the ingestion of those compounds [13]. Thus, foods containing higher levels of tryptophan and 5-hydroxytryptophan may help balance serotonin levels, a powerful antioxidant molecule. Cauliflowers are usually consumed after cooking, and in our study, the consumption of these cooked vegetables (steamed or microwaved) can be beneficial, since these processing methods do not allow the loss of these compounds to the cooking water. 

Steaming or microwaving increased the serotonin content in colored cauliflowers, with special highlight to ‘Verde di Macerata’ and ‘Forata’ cooked for 5 min (3.45 and 3.04 μg/g d.w., respectively). Interesting results were observed in the ‘Cheddar’ cauliflower microwaved for 10 min, which showed an increase of 76.53% in relation to the raw inflorescence. The serotonin results of ‘Graffiti’ do not show a significant difference after the thermal processing; however, presented the lower serotonin levels in relation to the other genotypes. Decreased levels of serotonin after thermal processing of fruits has been reported recently [5]. Nevertheless, in our study, the increased level of bioactive amines, as well as their precursors, may be resulted from food matrix disruption induced by the cooking process [25]. The thermal processes to which inflorescences were submitted probably facilitated the extraction of some amines (tryptamine, serotonin, melatonin, putrescine, spermidine, spermine and histamine) and their levels may be dependent on the molecule polarity, the matrix, the genotype and the cooking method [25]. 

**Figure 3:**
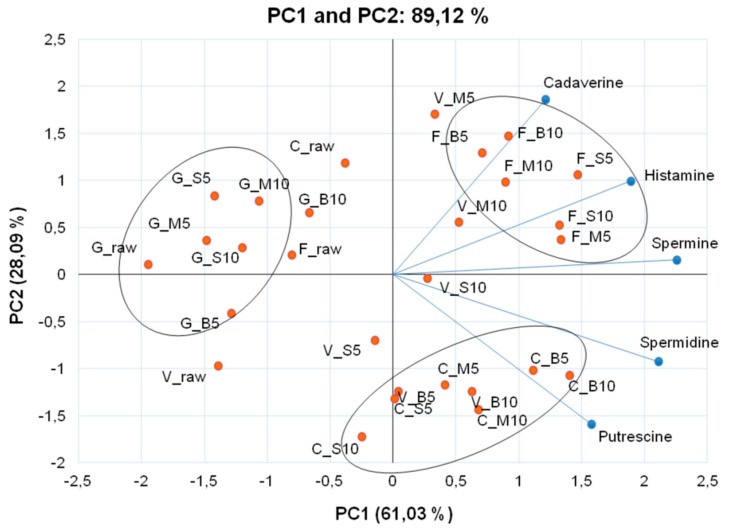
Bi-dimensional projection and biogenic amine scores (cadaverine, histamine, spermidine, spermine and putrescine) in thermally processed colored cauliflowers. The treatments are represented by the points, where initial letters represent the genotypes (V = ‘Verde di Macerata’, G = ‘Graffiti’, F = ‘Forata’ and C = ‘Cheddar’) and the letters after underline represent the cooking methods (raw, B = boiling, S = steam and M = microwave) and the numbers represent cooking times (5 and 10 min).

Comparing the colored cauliflowers, ‘Cheddar’ presented the highest levels of dopamine (Table 1) and was grouped into PC1+ (Figure 1). In this genotype, none of the thermal processes increased in dopamine levels (Table 1). On the other hand, ‘Verde di Macerata’ microwaved for 5 min showed the highest dopamine content, whereas the boiling of ‘Forata’ inflorescence, independent of the cooking time, promoted losses of this biogenic amine. Dopamine is considered a strong antioxidant when compared to other hydrophilic molecules, such as vitamin C and certain phenolic compounds when measured by DPPH [31]. This antioxidant activity is attributed to the o-dihydroxylated structure, and its amino acid residue increases the hydrophilic character [32]. Some amines are usually reported as quality markers of food storage, processing or degradation, especially tyramine, histamine, cadaverine and putrescine. In this study, tyramine levels were below the detection limit in all cauliflower genotypes. In order to compare the levels of histamine, cadaverine and putrescine, beyond spermidine and spermine in raw and cooked colored cauliflowers, we used the principal component analysis to visualize the results better (Figure 3). PC1 and PC2 explain 89.12% of the data, separating the genotypes and cooking methods used. All analyzed amines were grouped in PC1+, which explains 61.03% of the data.

Before thermal processing, the higher histamine content was observed in ‘Cheddar’, but this result changed after the cooking. Histamine was found in all genotypes, and the highest levels occurred in ‘Forata’ (Table 2), which was grouped in PC1+ (Figure 3), mainly after boiling for 10 min (0.71 μg/g d.w.). It should be pointed out that the levels of histamine in colored inflorescences are not considered to be harmful to health. Historically the illness caused by histamine has been known as “scombroid poisoning” because the high levels of histamine found in fish of the Scombridae and Scomberesocidae families. The United States Food and Drug Administration has recommended the limits of histamine consumption to 50 mg/kg of body weight [33]. However, in most cases, histamine levels necessary to cause food poisoning range from 200 ppm to 500 ppm [33]. In addition to histamine and tyramine, which are considered relevant for food safety, putrescine and cadaverine may potentiate the effect of histamine, by inhibiting histamine detoxifying enzymes [34]. In this study we noticed in ‘Forata’ and ‘Cheddar’, a strong correlation between histamine and cadaverine (*R*^2^ = 0.72 and *R*^2^ = 0.91, respectively). The highest levels of cadaverine were verified in ‘Cheddar’, which may explain the strong correlation found. However, thermal processing was beneficial, promoting a decrease in the content of this amine (Table 2). High levels of putrescine and cadaverine in food may indicate low quality, which makes it unsuitable for the consumption; so they are considered indicators or markers for the decomposition of unfermented foods [35].

The levels of cadaverine increased when ‘Forata’, ‘Verde di Macerata’ and ‘Graffiti’ cauliflowers were cooked, and the highest content was observed in ‘Forata’ boiled for 10 min (0.45 μg/g d.w.). The cooking process also induced increases in putrescine levels (PC1+) (Figure 3), mainly in ‘Cheddar’, reaching 12.04 μg/g d.w. after boiling for 10 min (Table 2). There was a poor correlation, either positive or negative, between putrescine and cadaverine levels in all genotypes (‘Verde de Macerata’, *R*^2^ = − 0.11; ‘Cheddar’, *R*^2^ = − 0.20; ‘Forata’, *R*^2^ = 0.21 and ‘Graffiti’, *R*^2^ = 0.29). Regarding cauliflower food safety, the putrescine and cadaverine levels are not considered to be harmful to health. For both, the level becomes toxic when the dose consumed is higher than 2000 mg/kg body weight [36]. Thus, to promote a negative effect on consumers would be necessary to ingest 1.7 kg of boiled ‘Cheddar’ inflorescences, where the highest levels of prutrescine and cadaverine were found. 

The spermidine and spermine levels (Table 2) increased considerably after the thermal processing in all genotypes. Both polyamines were grouped into PC1+. The lowest levels of all amines analyzed were observed in in ‘Graffiti’ (Figure 3) and they were grouped in PC1-. The boiling for 10 min was the cooking method that promoted the highest levels of spermine and spermidine in ‘Verde di Macerata’ and ‘Cheddar’. In both cooking methods, steam or microwave, ‘Forata’ showed higher spermidine and spermine content.

In addition to the positive effects already described, some biogenic amines may form nitrosamines, molecules related to cancer and mutagenic activity [37]. Spermidine and spermine are considered growth regulators, involved in the growth processes, intracellular signaling, cell division and differentiation [20], besides they play a role in protecting cells from free radicals’ action [38]. The spermidine contents were higher than those detected for spermine in all colored cauliflowers and the level considered toxic of both polyamines is 600 mg/kg of weight [39]. In our study, the intake of 100 g of boiled ‘Cheddar’ for 10 min, provide the ingestion of spermidine (3.74 mg/100 g d.w.) and spermine (0.53 mg/100 g d.w.) in levels considered not harmful to health. However, due to the action in cell division, is recommended that foods with higher levels of these amines (spermidine and spermine) should be avoided in patients in the treatment of certain neoplasm [40]. Interesting results were found in ‘Graffiti’, which showed the lowest levels of spermidine and spermine, and could be considered safe for individuals with diet restriction of biogenic amines. On the other hand, a recent study has shown that high spermidine intake is related to increased survival in humans [41]. According to our results, the levels of biogenic amines are depending on the genotype and the thermal processing, including the cooking time. Among the amines analyzed, some standout, such as histamine, putrescine and cadaverine, which are used as markers of food contamination by microorganisms [34]. There are certain controversies in the literature about the thermal stability of biogenic amines [5,42]. This behavior was observed in the samples analyzed, with a remarkable increasing tendency in these molecules after thermal processing. 

The CQI was calculated to verify food safety when cauliflowers are ingested raw or after cooking. This index is usually used for fishery products and consider values above 10 as indicators of food quality losses [21]. The highest CQI was observed in the genotype ‘Verde di Macerata’ without thermal processing; however, this value (1.17) is almost nine times lower than the maximum limit acceptable for consumption. In the meanwhile, all the other samples, raw or cooked, had CQI below the levels considered toxic for human consumption (Table 2). The CQI of biogenic amines may be a good tool to verify the quality and safety of vegetable consumption, especially after cooking, since thermal processing has a direct effect on these bioactive compounds. 

## 4. Conclusions

The cooking methods employed in this study resulted in different responses on the content of biogenic amines and amino acids, such as tryptophan and 5-hydroxytryptophan. After thermal processing, the levels of tryptophan were variable with a tendency to increase. Reduced levels of 5-hydroxytryptophan and increasing in serotonin were observed, especially after steaming and microwaving. ‘Forata’ showed the higher content of histamine after cooking, regardless of cooking method or time of processing; however, the quantities found are within the tolerable level of consumption and are not considered to be harmful to health. ‘Forata’ also show the higher melatonin content when steamed and microwaved in comparison to the other ones, which makes it a good source of this biogenic amine. The ‘Graffiti’ inflorescence presented the lowest levels of all bioactive amines, compared to the others cauliflowers studied, indicating that this genotype can be used in the diet of patients with some restriction to biogenic amines. All colored cauliflowers analyzed presented CQI below the pre-established limits, and did not represent a risk for consumers. The consumption of cauliflower steamed or microwaved, regardless of the preparation time (5 or 10 min), may be beneficial to human health and a good source of antioxidants (bioactive amines—melatonin, serotonin, dopamine, spermidine, and spermine). 

## Figures and Tables

**Table 1:** Amino acids (AA) and biogenic amines content (ug/g d.w.) in colored cauliflowers raw and thermally processed.

Cooking Methods	Time (min)	AA		Monoamines			
		Tryptophan	5-Hydroxytryptophan	Tryptamine	Serotonin	Melatonin	Dopamine
		**‘Verde di Macerata’**					
	**Raw**	79.30 ± 4.44 oF ^1^	254.39 ± 7.17 bA	5.18 ± 0.04 oE	0.87 ± 0.01 mD	0.40 ± 0.11 kB	0.08 ± 0.00 mD
**Boiling**	**5**	277.09 ± 13.33 fD	208.74 ± 13.42 cB	11.88 ± 0.17 bA	0.85 ± 0.03 mD	0.31 ± 0.04 kC	0.06 ± 0.01 nE
	**10**	354.91 ± 10.30 cB	70.05 ± 5.85 hE	8.18 ± 0.17 jC	0.81 ± 0.00 nD	0.31 ± 0.00 kC	0.22 ± 0.01 hB
**Steaming**	**5**	250.65 ± 7.18 gE	124.95 ± 1.35 eC	7.66 ± 0.10 kD	1.25 ± 0.04 kC	0.40 ± 0.02 kB	0.11 ± 0.00 lC
	**10**	302.67 ± 0.88 eC	128.81 ± 0.41 eC	8.53 ± 0.07 iB	1.29 ± 0.00 kC	0.59 ± 0.17 jA	0.08 ± 0.01 mD
**Microwave**	**5**	238.71 ± 0.64 hE	90.93 ± 1.26 fD	7.43 ± 0.01 lD	3.45 ± 0.14 cA	0.17 ± 0.03 kC	0.35 ± 0.00 cA
	**10**	432.37 ± 13.12 aA	136.40 ± 4.85 dC	8.69 ± 0.20 hB	1.41 ± 0.04 jB	0.50 ± 0.03 jA	0.03 ± 0.00 qF
		**‘Cheddar’**					
	**Raw**	337.35 ± 9.88 dC	384.97 ± 5.84 aA	9.00 ± 0.22 gD	1.00 ± 0.02 lF	5.33 ± 0.01 eC	0.42 ± 0.00 aA
**Boiling**	**5**	362.25 ± 2.60 cB	36.76 ± 1.23 lE	9.79 ± 0.05 fC	2.40 ± 0.01 hE	6.78 ± 0.08 dB	0.35 ± 0.01 bB
	**10**	399.40 ± 14.07 bA	45.93 ± 3.02 kD	11.53 ± 0.49 cB	2.71 ± 0.10 gD	4.29 ± 0.43 gD	0.24 ± 0.01 fD
**Steaming**	**5**	244.80 ± 5.28 hE	71.42 ± 2.54 hB	13.90 ± 0.34 aA	3.34 ± 0.09 dC	4.20 ± 0.13 gD	0.34 ± 0.01 dC
	**10**	301.35 ± 4.48 eD	70.39 ± 0.09 hB	8.74 ± 0.09 hD	3.70 ± 0.02 bB	5.21 ± 0.01 eC	0.22 ± 0.00 hE
**Microwave**	**5**	207.22 ± 2.26 iF	57.03 ± 3.55 iC	11.40 ± 0.16 cB	3.68 ± 0.14 bB	8.25 ± 0.16 cA	0.34 ± 0.01 dC
	**10**	211.49 ± 10.28 iF	55.90 ± 0.06 iC	8.63 ± 0.34 hD	4.26 ± 0.14 aA	4.48 ± 0.26 gD	0.13 ± 0.01 kF
		**‘Forata’**					
	**Raw**	36.53 ± 0.56 pF	203.25 ± 0.72 cA	4.69 ± 0.03 pE	2.03 ± 0.02 hC	0.97 ± 0.05 iE	0.12 ± 0.01 kD
**Boiling**	**5**	167.97 ± 3.42 lE	45.50 ± 0.56 kE	8.60 ± 0.15 hD	1.86 ± 0.05 iD	4.79 ± 0.32 fC	0.07 ± 0.00 mE
	**10**	184.81 ± 2.83 kD	45.81 ± 1.30 kE	8.84 ± 0.11 gC	1.81 ± 0.10 iD	4.28 ± 0.02 gD	0.05 ± 0.00 oF
**Steaming**	**5**	169.47 ± 1.58 lE	66.83 ± 0.81 hB	10.26 ± 0.10 eB	2.83 ± 0.02 gB	9.34 ± 0.26 aA	0.13 ± 0.00 kD
	**10**	256.65 ± 2.21 gA	49.66 ± 1.71 jD	10.89 ± 0.16 dA	2.79 ± 0.02 gB	9.02 ± 0.04 bA	0.28 ± 0.01 eA
**Microwave**	**5**	216.63 ± 2.37 iC	52.68 ± 1.37 jC	10.40 ± 0.02 eB	3.04 ± 0.06 eA	8.39 ± 0.00 cB	0.23 ± 0.00 gB
	**10**	235.09 ± 1.22 hB	42.50 ± 0.63 kF	10.48 ± 0.07 eE	2.91 ± 0.04 fB	9.25 ± 0.11 aA	0.15 ± 0.00 jC
		**‘Graffiti’**					
	**Raw**	21.29 ± 0.06 qG	42.90 ± 0.42 kC	5.46 ± 0.01 nG	0.45 ± 0.00 oB	1.06 ± 0.12 iB	0.18 ± 0.00 iA
**Boiling**	**5**	175.98 ± 0.29 kB	27.78 ± 2.10 mE	7.72 ± 0.16 kC	0.75 ± 0.06 nA	0.49 ± 0.04 jD	0.04 ± 0.00 pC
	**10**	193.56 ± 2.86 jA	27.57 ± 0.17 mE	6.28 ± 0.04 mF	0.70 ± 0.02 nA	0.46 ± 0.09 jD	0.05 ± 0.00 oB
**Steaming**	**5**	87.45 ± 0.33 oF	42.33 ± 0.13 kC	7.55 ± 0.03 kD	0.81 ± 0.09 nA	0.94 ± 0.04 iB	0.02 ± 0.00 rD
	**10**	159.22 ± 2.02 mD	50.49 ± 1.97 jB	7.24 ± 0.06 lE	0.80 ± 0.01 nA	0.90 ± 0.02 iB	0.02 ± 0.00 rC
**Microwave**	**5**	147.81 ± 0.59 nE	37.11 ± 1.65 lD	8.48 ± 0.08 iB	0.73 ± 0.00 nA	0.68 ± 0.03 jC	0.02 ± 0.00 rD
	**10**	165.08 ± 0.42 lC	77.13 ± 0.11 gA	10.37 ± 0.02 eA	0.77 ± 0.01 nA	1.63 ± 0.26 hA	0.02 ± 0.00 rD

^1^ Results are expressed as mean ± standard deviation (*n* = 3). The averages followed by the same lowercase letter (all treatments) and uppercase letter (genotypes) do not differ statistically from each other. The Scott-Knott’s test was applied at the 5% probability level.

**Table 2:** Monoamine, diamines, polyamines (ug/g d.w.) and CQI in colored cauliflowers raw and thermally processed.

Cooking Methods	Time (min)	Monoamine	Diamines		Polyamines		CQI
		Histamine	Cadaverine	Putrescine	Spermidine	Spermine	
		**‘Verde di Macerata’**					
	**Raw**	0.06 ± 0.00 kE ^1^	0.03 ± 0.00 nF	3.58 ± 0.05 iE	1.84 ± 0.04 nG	0.29 ± 0.02 pE	1.17 ± 0.03
**Boiling**	**5**	0.10 ± 0.00 jD	0.08 ± 0.03 kE	5.87 ± 0.10 fC	14.86 ± 0.31 gB	1.83 ± 0.02 jB	0.34 ± 0.00
	**10**	0.12 ± 0.00 jD	0.10 ± 0.01 jE	7.68 ± 0.06 dA	21.38 ± 0.22 cA	3.08 ± 0.03 iA	0.31 ± 0.00
**Steaming**	**5**	0.11 ± 0.01 jD	0.12 ± 0.01 iD	6.24 ± 0.07 eB	13.29 ± 0.17 iD	1.37 ± 0.02 lD	0.41 ± 0.00
	**10**	0.21 ± 0.02 iC	0.18 ± 0.01 gC	4.49 ± 0.03 gD	13.66 ± 0.09 iC	1.68 ± 0.01 kC	0.30 ± 0.00
**Microwave**	**5**	0.56 ± 0.01 cA	0.39 ± 0.02 bA	1.45 ± 0.03 lF	9.74 ± 0.01 kF	1.81 ± 0.01 jB	0.19 ± 0.00
	**10**	0.37 ± 0.02 gB	0.31 ± 0.01 cB	5.95 ± 0.13 fC	11.54 ± 0.26 jE	1.69 ± 0.04 kC	0.47 ± 0.00
		**‘Cheddar’**					
	**Raw**	0.46 ± 0.02 eA	0.27 ± 0.02 dA	2.04 ± 0.03 kD	3.50 ± 0.02 mF	0.35 ± 0.02 pF	0.57 ± 0.01
**Boiling**	**5**	0.41 ± 0.01 fB	0.09 ± 0.00 kC	7.71 ± 0.06 dC	27.70 ± 0.13 bB	4.15 ± 0.00 fB	0.25 ± 0.00
	**10**	0.48 ± 0.05 eA	0.10 ± 0.01 nB	12.04 ± 0.48 aA	37.44 ± 1.45 aA	5.29 ± 0.17 eA	0.29 ± 0.00
**Steaming**	**5**	0.22 ± 0.02 iD	0.03 ± 0.00 nE	7.56 ± 0.20 dC	13.54 ± 0.33 iD	1.37 ± 0.03 lD	0.49 ± 0.00
	**10**	0.20 ± 0.01 iD	0.02 ± 0.00 nE	8.90 ± 0.10 bB	11.74 ± 0.13 jE	0.77 ± 0.01 nE	0.68 ± 0.00
**Microwave**	**5**	0.27 ± 0.01 hC	0.07 ± 0.00 lD	8.08 ± 0.13 cC	14.08 ± 0.28 hD	1.42 ± 0.02 lD	0.51 ± 0.00
	**10**	0.27 ± 0.05 hC	0.07 ± 0.00 lD	8.96 ± 0.42 bB	16.87 ± 0.55 fC	1.89 ± 0.05 jC	0.47 ± 0.00
		**‘Forata’**					
	**Raw**	0.04 ± 0.00 kD	0.20 ± 0.00 fE	3.22 ± 0.02 jF	4.60 ± 0.05 lG	0.85 ± 0.01 nG	0.54 ± 0.00
**Boiling**	**5**	0.60 ± 0.06 bB	0.28 ± 0.02 dC	3.37 ± 0.07 jD	10.29 ± 0.17 kE	4.04 ± 0.06 gE	0.28 ± 0.00
	**10**	0.71 ± 0.01 aA	0.45 ± 0.02 aA	4.24 ± 0.02 hB	10.02 ± 0.09 kF	3.87 ± 0.03 hF	0.36 ± 0.00
**Steaming**	**5**	0.65 ± 0.04 bA	0.32 ± 0.00 cB	3.48 ± 0.05 iC	20.58 ± 0.20 dA	7.60 ± 0.08 bB	0.15 ± 0.00
	**10**	0.60 ± 0.06 bB	0.24 ± 0.00 eD	4.16 ± 0.04 hB	19.11 ± 0.24 eB	8.17 ± 0.11 aA	0.18 ± 0.00
**Microwave**	**5**	0.62 ± 0.03 bB	0.21 ± 0.00 fE	4.64 ± 0.06 gA	18.73 ± 0.04 eC	6.98 ± 0.01 cC	0.20 ± 0.00
	**10**	0.52 ± 0.03 dC	0.27 ± 0.01 dC	3.32 ± 0.01 jE	14.02 ± 0.08 hD	5.80 ± 0.03 dD	0.20 ± 0.00
		**‘Graffiti’**					
	**Raw**	0.09 ± 0.00 kC	0.05 ± 0.00 mD	0.34 ± 0.01 nF	0.52 ± 0.00 pD	0.13 ± 0.01 qE	0.29 ± 0.00
**Boiling**	**5**	0.11 ± 0.01 jB	0.03 ± 0.01 nE	0.96 ± 0.01 mB	1.46 ± 0.01 oB	0.82 ± 0.02 nB	0.34 ± 0.01
	**10**	0.13 ± 0.01 jA	0.16 ± 0.02 hA	1.38 ± 0.02 lA	1.91 ± 0.25 nA	1.10 ± 0.03 mA	0.42 ± 0.02
**Steaming**	**5**	0.12 ± 0.00 jA	0.10 ± 0.00 jB	0.71 ± 0.01 mE	0.63 ± 0.05 pD	0.33 ± 0.02 pD	0.48 ± 0.01
	**10**	0.13 ± 0.00 jA	0.05 ± 0.00 mD	0.78 ± 0.01 mD	1.36 ± 0.02 oB	0.56 ± 0.00 oC	0.33 ± 0.00
**Microwave**	**5**	0.11 ± 0.00 jB	0.09 ± 0.00 kC	0.81 ± 0.01 mC	0.68 ± 0.02 pD	0.32 ± 0.02 pD	0.51 ± 0.00
	**10**	0.13 ± 0.01 jA	0.11 ± 0.01 jB	0.83 ± 0.00 mC	1.20 ± 0.00 oC	0.55 ± 0.00 oC	0.39 ± 0.01

^1^ Results are expressed as mean ± standard deviation (*n* = 3). The averages followed by the same lowercase letter all treatments) and uppercase letter (genotypes) do not differ statistically from each other. The Scott-Knott’s test was applied at the 5% probability level.
